# A non-invasive modifiable Healthy Ageing Nutrition Index (HANI) predicts longevity in free-living older Taiwanese

**DOI:** 10.1038/s41598-018-24625-3

**Published:** 2018-05-08

**Authors:** Yi-Chen Huang, Mark L. Wahlqvist, Yuan-Ting C. Lo, Chin Lin, Hsing-Yi Chang, Meei-Shyuan Lee

**Affiliations:** 1 Department of Nutrition, China Medical University, 91 Hsueh-shih Road, Taichung, 40402 Taiwan, ROC; 2 Graduate Institute of Life Sciences, National Defense Medical Center, 161 Minchuan East Road, Sec. 6, Taipei, 11490 Taiwan, ROC; 3 Institute of Population Health Sciences, National Health Research Institutes, 35 Keyan Road, Zhunan, Miaoli County 35053 Taiwan; 4 School of Public Health, National Defense Medical Center, 161 Minchuan East Road, Sec. 6, Taipei, 11490 Taiwan, ROC; 5 Monash Asia Institute, Monash University, 900 Dandenong Road, Caulfield East, Melbourne, Victoria 3145 Australia; 6 Department of Research and Development, National Defense Medical Center, 161 Minchuan East Road, Sec. 6, Taipei, 11490 Taiwan, ROC

## Abstract

Nutritional factors contributing to disability and mortality are modifiable in later life. Indices would add utility. We developed a gender-specific Healthy Ageing Nutrition Index (HANI) for all-cause mortality in free-living elderly. We stratified 1898 participants aged ≥65 y from the 1999–2000 Nutrition and Health Survey in Taiwan by region and randomly allocated them into development and validation sets. Linkage to the National Death Registry database until December 31, 2008 enabled mortality prediction using Cox proportional-hazards models. Four factors (appetite, eating with others, dietary diversity score, and BMI) with best total of 25 HANI points for men; and 3 factors (cooking frequency, dietary diversity score, and BMI) with best total of 27 HANI points for women, were developed. In the validation set, the highest HANI group exhibited a greater intake of plant-derived food and associated nutrients, a favourable quality of life, and more muscle mass, compared with the lowest group. The highest HANI group predicts mortality risk lower by 44 percent in men and 61 percent in women. Adjusted mortality HRs were comparable between sets. HANI is a simple, non-invasive, inexpensive, and potentially modifiable tool for nutrition monitoring and survival prediction for older adults, superior to its individual components.

## Introduction

Population Ageing is a critical concern for global public health, because of its varied rates of increase and quality^1^. Quality of life (QOL) and disability compromise the value and extent of longevity and the majority of health expenditure is incurred in the last year of life^2^. Time trends which demonstrate reductions in age-specific disability provide evidence of avoidability^3^. To reduce the burden of disease and increase life expectancy in later life, the focus has been on chronic disease with its behavioural, social, and environmental contributors^4–6^. Some modifiable contributors include diet, physical activity, and substance abuse affecting well-being, disability, disease and survival^4,7–11^.

Diet-related factors are a leading cause of death globally^12–14^. Ageing is affected by demographic, epidemiologic, and nutritional transitions^15^ with nutrition-related factors the most modifiable. Nutritional factors derive from the underlying food system which is reflected in the food supply and shopping^16^, nutritional knowledge^17^, skills such as cooking^18^, along with dietary patterns and quality^9,19^. There are age-related nutritional conditions such as the anorexia of ageing^20,21^, chewing disability^22^, cognitive impairment^23^ and problems with food and eating^24^.

The marked differences in health and survival between genders might be partly attributed to nutritional status^25,26^. These might be on account of women tending to exhibit more health-seeking behavior^26,27^, higher apparent morbidity, and a higher use of health care services^28^ than men. For example, the consumption of fruits and vegetables in older people is affected by different social support forms between genders^29^. However, few tools screen for nutritional risk factors according to gender.

In this study, we identify modifiable nutritional- or diet-related risk factors for mortality in the older Taiwanese population as well as develop and validate a composite non-invasive gender-specific Healthy Ageing Nutrition Index (HANI) for survival prediction.

## Results

Measurement and distribution (%) of each candidate factor was presented in Table 1. Men more commonly reported good appetite, greater shopping frequency, greater physical activity, unsatisfactory chewing ability, and less cooking frequency compared to women (*P* < 0.05).

**Table 1:** Measurement and distribution (%) of each candidate factor by gender. USD, US Dollar; NTD, Taiwan New Dollar; METs, metabolic equivalents.

Candidate factors	Measurement or question	Full cohort	Men (n = 970)			Women (n = 967)			*P* value
			All	Development	Validation	All	Development	Validation	
Appetite status	How is your current appetite?								0.01
Poor		10.2	8.06	7.54	8.58	12.5	12.4	12.5	
Fair		56.2	54.2	51.5	56.9	58.4	59.0	57.8	
Good		33.6	37.8	41.0	34.5	29.2	28.6	29.7	
Satisfactory chewing	Do you have difficulty in chewing food? (yes/no)	38.7	34.5	37.0	32.1	43.2	43.9	42.5	0.002
Dietary diversity score ≤3 4 5 6	Measured by a 24-hour dietary recall of 6 food groups or by asking the following question. “Did you eat more than half a serving size of any of the following foods yesterday?” • Breads, cereals, starches (e.g., bread 1 slice, cereal 1/2 cup, bagels 1/2, white rice 1/2 cup) • Dairy (e.g., milk 1/2 cup, yogurt 50 g, cheese 1 slice) • Meat, fish, egg or legumes (e.g., 1.5 oz cooked meat, egg 1/2, soymilk 1/2 cup) • Vegetables (e.g., 1/2 cup) • Fruits (e.g., oranges 1/2, apples 1/2, pears 1/2, bananas 1/2) • Fats and oils (e.g., 1/2 table spoon)								0.151
		17.7	15.9	15.5	16.2	19.7	20.3	19.1	
		30.6	32.0	30.4	33.5	29.2	29.8	28.5	
		34.5	35.1	36.6	33.6	33.8	34.7	32.9	
		17.2	17.1	17.5	16.7	17.4	15.2	19.5	
Vegetable expenditure, NTD/day	Vegetable food cost derived from a 24-hour dietary recall (1 USD ≈ 32 NTD)								0.332
<10.07		27.1	28.4	29.4	27.3	25.6	24.0	27.2	
10.07–<18.76		26.6	26.4	28.6	24.2	26.9	25.5	28.3	
18.76–<26.05		19.7	20.2	19.0	21.4	19.1	21.0	17.3	
≥28.05		26.6	25.1	23.1	27.1	28.3	29.5	27.2	
Cooking frequency	Do you have to cook or prepare food for yourself or help with these? Do not include ready-to-eat food.								<0.0001
Never (<1/month)		43.1	58.5	56.7	60.4	26.5	28.2	24.8	
Sometimes		17.0	20.4	20.6	20.1	13.4	13.1	13.7	
Often		8.56	6.70	8.40	4.99	10.6	10.3	10.8	
Usually		31.3	14.4	14.4	14.5	49.6	48.5	50.7	
Eat with others	Do you eat with others at least one meal a day? (yes/no)	83.9	85.7	86.4	85.0	82.0	81.9	82.1	
									0.125
Alcohol drinking	Do you drink alcoholic beverage? (yes/no)	18.3	30.8	29.9	31.7	4.75	5.78	3.72	<0.0001
Shopping frequency	What is the frequency with which you go out shopping?								0.002
<1/week		49.2	44.3	43.0	45.6	54.5	51.8	57.3	
1/week		13.0	12.5	12.7	12.2	13.5	16.2	10.9	
2–4/week		21.4	22.9	22.1	23.7	19.7	20.7	18.8	
Everyday		16.5	20.4	22.2	18.6	12.2	11.4	13.0	
Physical activity, METs/day	Number of METs per day for leisure time physical activity								0.0004
<1.5		56.3	51.6	48.6	54.6	61.3	61.3	61.2	
1.5–2.9		11.4	10.9	11.3	10.6	11.9	11.7	12.2	
≥3		32.3	37.4	40.1	34.8	26.8	27.0	26.6	
Body mass index, kg/m^2^	Weight (kg)/[height(m)]^2^								<0.0001
<18.5		5.17	5.32	4.45	6.20	5.00	4.31	5.68	
18.5–23.9		54.0	63.5	65.4	61.7	43.7	49.1	38.4	
24.0–26.9		28.4	21.4	20.9	21.9	35.9	31.4	40.3	
≥27.0		12.5	9.72	9.25	10.2	15.4	15.2	15.6	
Recommended waist circumference	Men: <90 cm; women: <80 cm	58.4	77.0	76.7	77.2	38.2	40.4	36.1	<0.0001

### HANI development

We first selected 8 factors for men and 7 for women with age-adjusted HRs ≤ 1. Then, according to the optimal fully adjusted gender-specific models with *P* < 0.05, we selected 4 factors for men and 2 for women. Dietary diversity score (DDS) was added based on a literature review for women. For men, each score for selected factors to HANI were appetite [9], eating with others [2], DDS [7], and body mass index (BMI) [7], and for women, they were frequency of cooking [11], DDS [7], and BMI [9]. The total HANIs were 25 for men and 27 for women (Table 2).

**Table 2:** Hazard ratios (95% confidence interval) of selected variables in the development set for HANI.

	Men (n = 474)					Women (n = 471)			β	Scoring (27)
	Age adjusted model^a^	Fully adjusted model (Selected variables HR < 1)		β	Scoring (25)	Age adjusted model^a^	Fully adjusted model (Selected variables HR <1)			
	HR (95% CI)	HR (95% CI)	*P* value			HR (95% CI)	HR (95% CI)	*P* value		
Appetite status			0.0002					0.94		
Poor	1	1		0	0	1	1			
Fair	0.28 (0.15–0.55)	0.34 (0.18–0.63)		−1.30	7	0.67 (0.36–1.27)	0.96 (0.45–2.07)			
Good	0.18 (0.09–0.38)	0.23 (0.13–0.42)		−1.62	9	0.53 (0.28–1.00)	0.90 (0.46–1.77)			
Satisfactory chewing	0.76 (0.45–1.30)	0.84 (0.50–1.40)	0.49			0.61 (0.34–1.08)	0.59 (0.33–1.06)	0.074		
Dietary diversity score			0.0003					0.20		
≤3	1	1		0	0	1	1		0	0
4	0.44 (0.28–0.69)	0.41 (0.24–0.70)		−0.79	4	1.05 (0.53–2.08)	0.78 (0.42–1.44)		−0.13	1
5	0.31 (0.19–0.51)	0.29 (0.16–0.52)		−1.07	6	0.79 (0.42–1.51)	0.55 (0.29–1.03)		−0.54	4
6	0.26 (0.13–0.52)	0.23 (0.11–0.46)		−1.40	7	0.61 (0.22–1.71)	0.43 (0.18–1.04)		−0.91	7
Vegetable expenditure, NTD/day			0.21					0.25		
<10.07	1	1				1	1			
10.07–<18.76	1.08 (0.73–1.59)	0.93 (0.64–1.36)				0.84 (0.42–1.68)	0.61 (0.29–1.28)			
18.76–<26.05	0.64 (0.32–1.28)	0.50 (0.24–1.04)				1.23 (0.54–2.81)	1.10 (0.56–2.14)			
≥28.05	0.84 (0.54–1.32)	0.70 (0.43–1.16)				0.83 (0.42–1.64)	0.77 (0.45–1.33)			
Cooking frequency								0.0002		
Never (<1/month)	1					1	1		0	0
Sometimes	1.27 (0.73–2.21)					0.63 (0.30–1.33)	0.63 (0.29–1.35)		−0.59	5
Often	1.17 (0.67–2.01)					0.74 (0.31–1.77)	0.67 (0.28–1.59)			
Usually	1.07 (0.56–2.03)					0.34 (0.20–0.57)	0.30 (0.18–0.50)		−1.37	11
Eating with others	0.59 (0.37–0.95)	0.61 (0.40–0.96)	0.03	−0.32	2	1.11 (0.71–1.75)				
Alcohol drinking	1.12 (0.73–1.70)					0.52 (0.21–1.25)	0.50 (0.24–1.04)	0.06		
Shopping frequency			0.14					0.69		
<1/week	1	1				1	1			
1/week	0.98 (0.59–1.65)	0.97 (0.57–1.64)				0.89 (0.47–1.66)	0.82 (0.42–1.60)			
2–4/week	0.92 (0.53–1.61)	0.73 (0.43–1.25)				0.87 (0.47–1.66)	0.95 (0.53–1.70)			
Everyday	0.70 (0.44–1.11)	0.61 (0.38–0.97)				0.47 (0.25–0.89)	0.69 (0.35–1.34)			
Physical activity, METs/day			0.45					0.41		
<1.5	1	1				1	1			
1.5–2.9	0.80 (0.42–1.51)	0.72 (0.38–1.36)				0.72 (0.34–1.51)	0.89 (0.42–1.90)			
≥3	0.68 (0.45–1.02)	0.80 (0.52–1.25)				0.70 (0.39–1.26)	0.68 (0.39–1.20)			
Body mass index, kg/m^2^			0.001					0.03		
<18.5	1	1		0	0	1	1		0	0
18.5–23.9	0.80 (0.32–1.99)	0.85 (0.36–2.03)		−0.19	1	1.18 (0.44–3.14)	0.76 (0.35–1.67)		−0.42	3
24.0–26.9	0.56 (0.19–1.63)	0.43 (0.13–1.36)		−0.80	4	0.70 (0.24–2.02)	0.36 (0.14–0.92)		−1.20	9
≥27.0	0.34 (0.10–1.16)	0.25 (0.08–0.85)		−1.38	7	0.80 (0.29–2.15)	0.35 (0.14–0.88)			
Recommended waist circumference	1.22 (0.84–1.76)					1.24 (0.84–1.83)				

Data were weighted for unequal probability of sampling design by SUDAAN and estimated Hazard ratio (95% confidence interval) by using the Cox proportional hazard model.

^a^Adjusted for age for each variable. HANI: Healthy Ageing Nutrition Index; HR, hazard ratio; NTD, Taiwan New Dollar; METs, metabolic equivalents.

**Figure 1:**
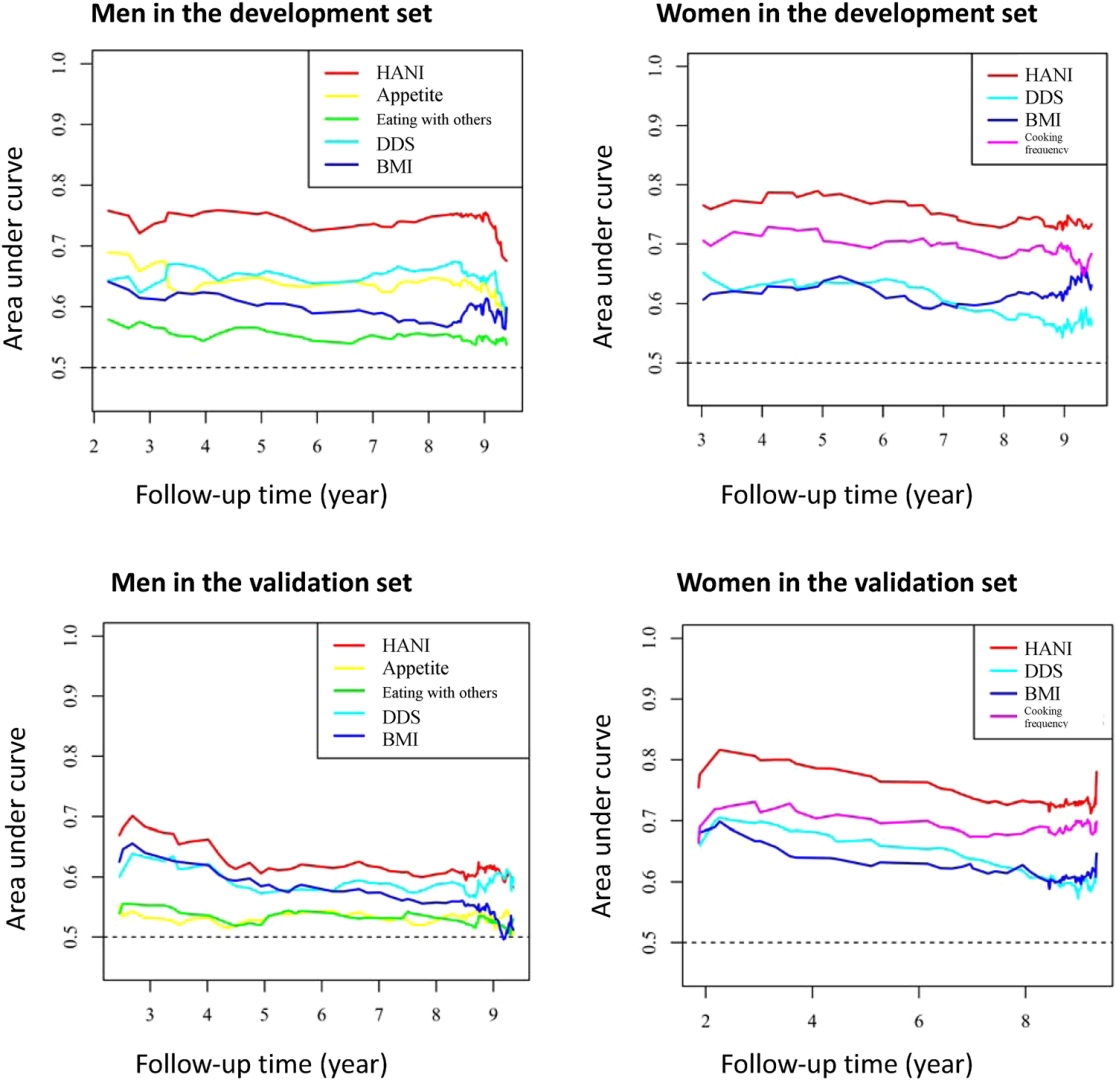
Area under curve of time-dependent receiver operating characteristic for HANI score and each component. HANI, Healthy Ageing Nutrition Index; DDS, dietary diversity score; BMI: body mass index.

For both men and women, the predictability over time by area under curve (AUC) of HANI was higher (around 0.7–0.8) than for its individual components (around 0.5–0.7) (Fig. 1). The C-statistics were 0.78 for men and 0.76 for women in the development set. We obtained similar statistics for the validation set (men = 0.70; women = 0.77) and the entire study cohort (men = 0.73; women = 0.75) (data not shown).

### Characteristics in the validation set

Both men and women with the highest HANI tended to be younger and live in an urban area, have a higher education level, higher social engagement, less cognitive impairment, and less difficulty in activities of daily living (ADL). (Table 3).

**Table 3:** Baseline characteristics of NAHSIT Elderly by the HANI in the validation set

Variables	Men^a^					Women^a^				
	Total	<14	14–16	>16	*P* value ^b^	Total	<14	14–20	>20	*P* value ^b^
n	483	114	170	199		470	138	185	147	
Weight n	632390	150700	219574	262116		588901	199495	200192	189215	
Total, %		23.8	34.7	41.5			33.9	34.0	32.1	
Cumulative death rate, per 1,000 personal year		96.4	53.2	44.4			78.3	37.8	22.0	
Age at baseline (y), %					0.015					0.0003
65–69	35.2	22.9	40.3	37.9		34.5	17.3	34.9	52.2	
70–74	29.6	30.0	25.3	33.0		28.1	19.7	32.6	32.3	
75–79	19.6	20.2	18.7	19.9		20.1	22.6	26.2	11.2	
≥80	15.7	26.9	15.8	9.26		17.2	40.5	6.24	4.31	
Education, %					0.002					<0.0001
Illiterate	18.5	19.7	27.8	10.1		55.8	77.2	49.1	40.3	
Some up to primary school	45.2	50.7	45.1	42.2		32.9	16.8	40.7	41.5	
High school and above	36.3	29.6	27.1	47.7		11.3	5.98	10.2	18.2	
Ethnicity, %					0.001					0.002
Fukienese	56.3	58.9	71.8	41.9		75.8	82.6	71.0	73.6	
Hakka	11.5	8.67	11.1	13.6		16.0	13.2	19.1	15.4	
Mainlander	30.7	29.9	16.0	43.3		6.83	3.10	7.35	10.2	
Aboriginal	1.47	2.49	1.07	1.22		1.47	1.11	2.5	0.76	
Region, %					0.0001					0.003
Hakka	10.3	7.33	7.23	11.2		11.4	9.82	15.0	9.25	
Mountainous areas	0.71	1.29	0.43	0.61		0.82	0.74	1.22	0.47	
Eastern	2.30	2.49	2.58	1.95		1.26	0.97	1.91	0.88	
Penghu	0.86	0.83	1.16	0.63		1.29	1.09	1.62	1.14	
Northern 1	14.5	23.7	3.69	18.3		17.7	9.92	16.9	26.7	
Northern 2	12.0	8.40	13.9	12.4		9.63	6.96	12.4	9.48	
Northern 3	9.72	13.6	10.6	6.76		7.83	7.62	8.17	7.69	
Central 1	7.12	5.32	8.56	6.96		7.80	12.4	4.39	6.54	
Central 2	8.24	7.65	6.23	10.3		9.18	12.9	7.61	6.89	
Central 3	6.37	5.63	6.41	6.76		6.49	6.10	7.61	5.73	
Southern 1	6.30	5.63	4.81	7.94		6.89	7.19	5.37	8.19	
Southern 2	8.07	6.36	8.69	8.54		6.21	8.38	4.87	5.34	
Southern 3	13.6	11.7	21.8	7.74		13.5	15.9	12.9	11.7	
Whether enough money, %					0.012					0.120
More than enough	75.0	56.5	78.3	82.5		76.1	77.4	70.2	81.1	
Just enough	23.3	39.8	20.7	16.2		19.9	16.1	25.2	18.2	
Not enough	1.77	3.72	1.02	1.29		4.00	6.60	4.60	0.66	
Smoker, %	66.8	74.9	69.6	59.9	0.018	4.79	5.69	3.07	5.65	0.495
Live alone, %	11.0	29.4	10.8	4.33	0.013	10.2	2.90	12.6	11.3	0.003
Pay or unpaid job, %	16.8	12.1	18.9	17.7	0.306	9.15	3.9	11.2	12.5	0.028
Less social engagement, %	8.05	22.2	3.39	3.81	0.030	12.1	23.7	10.1	1.88	0.0003
Physical activity (METs/day), %					0.063					0.0001
<1.5	54.1	60.1	61.5	44.6		61.3	73.6	64.7	44.8	
1.5–2.9	10.7	11.0	8.44	12.5		11.9	11.7	8.20	16.0	
≥3	35.1	28.8	30.1	43.0		26.8	14.8	27.1	39.2	
History of hypertension, %	37.2	41.8	29.0	41.4	0.090	39.8	36.8	37.5	45.3	0.500
History of diabetes, %	9.71	9.85	9.26	10.0	0.978	11.0	11.6	10.9	10.5	0.968
History of stroke, %	6.22	8.99	4.20	6.36	0.338	6.30	9.20	6.62	2.91	0.056
History of renal disease, %	2.96	1.21	3.18	3.76	0.241	2.62	2.39	1.97	3.56	0.704
History of cancer, %	2.22	3.8	2.48	1.12	0.538	2.5	2.12	2.03	3.36	0.761
Cognitive impairment, %	10.3	19.8	11.4	3.99	0.020	27.2	48.2	22.1	11.0	0.0001
ADL ≥1, %	6.91	15.7	6.69	2.06	0.013	12.1	29.0	5.71	1.04	0.0001
Charlson comorbidity index										
Mean	4.65	4.70	4.79	4.53	0.876	4.78	4.88	4.82	4.65	0.809
SE	0.20	0.56	0.43	0.32		0.21	0.39	0.35	0.29	

All data weighted for unequal probability of sampling design by SUDAAN. ^a^Cut-off points for HANI were based on Youden index. ^b^ANOVA and chi-square were used for continuous and categories variables to test difference between the HANI groups by gender. NAHSIT, Nutrition and Health Survey in Taiwan; HANI, Healthy Ageing Nutrition Index; METs, metabolic equivalents; ADL, activities of daily living; SE, standard error.

Men with HANI > 16 had a higher daily frequency of dairy (0.82 vs. 0.46), vegetable (2.54 vs. 1.84), fruit (1.34 vs. 0.98) and total energy intakes (2067 vs. 1534 kcal/d) compared with those with HANI < 14. However, in women, only fruit intake was higher (1.43 vs. 0.87). For nutrient intakes, both men and women with the highest HANI tended to consume more vitamin C and calcium. Women also consumed more dietary fiber (15.3 vs. 12.0 g/d), vitamin B-2 (1.16 vs. 0.77 mg/d), magnesium (175 vs. 150 mg/d), and potassium (1746 vs. 1425 mg/d) than did those with HANI < 14. (Supplementary Table S1).

**Figure 2:**
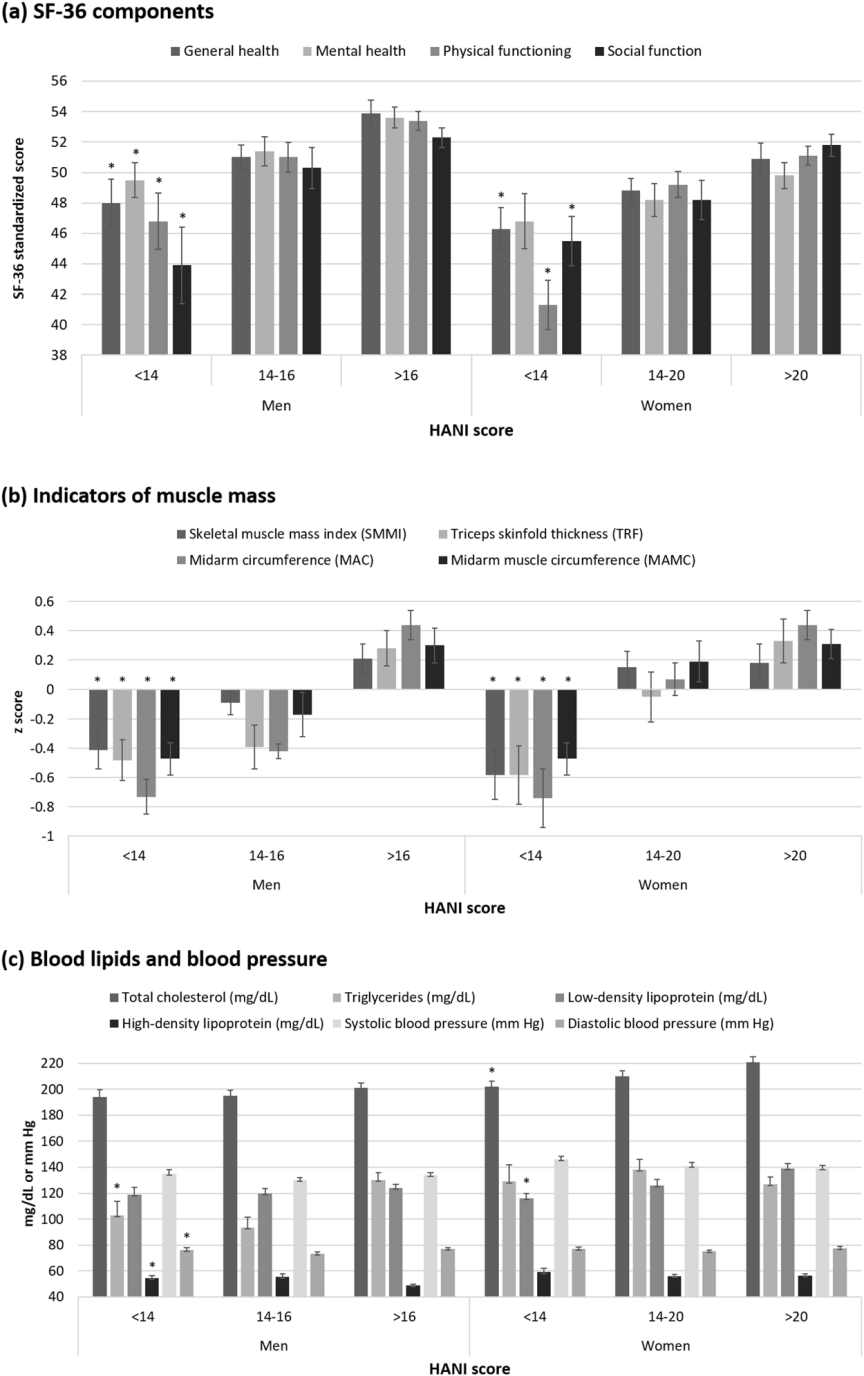
Mean of SF-36 components (**a**), indicators of muscle mass (**b**) and (**c**) blood lipids as well as blood pressure between HANI groups by gender in the validation set. *P* values were examined by ANOVA. ^*^P value less than 0.05 between HANI groups in men and women, respectively. HANI, Healthy Ageing Nutrition Index; SF-36, Short Form-36.

Both men and women with the highest HANI had higher scores for each component of SF-36 and higher muscle mass (skeletal muscle mass index (SMMI), mid-arm circumference (MAC), mid-arm muscle circumference (MAMC)), and body fat (triceps skinfold thickness (TSF)). For the cardiometabolic biomarkers, men with HANI > 16 had higher triglycerides and lower high-density lipoprotein (HDL) than did those with HANI < 14. Women with the highest HANI had higher total cholesterol and low-density lipoprotein (LDL). (Fig. 2).

### HANI and all-cause mortality

In the development and validation sets, men with the highest HANI had a lower risk of all-cause mortality (hazard ratio (HR): 0.15, 95% confidence interval (CI): 0.09–0.26; HR: 0.45, 95% CI: 0.32–0.64, respectively), compared with those with the lowest HANI. (Table 4) After adjustment for age, region, education, smoking status, physical activity (PA), and social engagement (model 2), the HRs (95% CI) for men with HANIs of 14–16 and >16 were 0.39 (0.27–0.58) and 0.20 (0.12–0.35), respectively, in the development set and 0.57 (0.37–0.86) and 0.61 (0.38–0.97), respectively, in the validation set (*P* < 0.05). The risk of all-cause mortality was reduced by 27% (HR: 0.73, 95% CI: 0.66–0.80) and 8% (HR: 0.92, 95% CI: 0.85–1.01), with a 2-point increase in HANI, in the development and validation sets, respectively.

**Table 4:** Hazard ratios (95% confidence interval) for the association between HANI and risk of all–cause mortality in NAHSIT Elderly by gender.

	HANI, hazard ratios (95% confidence interval)									
	Men					Women				
	<14	14–16	>16	*P* for trend	2 point increase	<14	14–20	>20	*P* for trend	2 point increase
Full cohort										
Deceased/survival, n	144/80	135/205	109/284			154/141	107/265	51/223		
Years of follow-up, median	6.37	8.72	8.86			8.18	8.92	8.95		
Cumulative death rate, per 1,000 personal year	112	54.8	35.0			81.1	36.5	22.4		
Crude model	1.00	0.41 (0.31–0.56)	0.27 (0.19–0.39)	<0.0001	0.77 (0.72–0.83)	1.00	0.41 (0.29–0.59)	0.22 (0.13–0.35)	<0.0001	0.45 (0.35–0.58)
Model 1	1.00	0.45 (0.33–0.61)	0.31 (0.22–0.45)	<0.0001	0.80 (0.74–0.85)	1.00	0.64 (0.46–0.89)	0.38 (0.23–0.64)	0.0007	0.62 (0.48–0.80)
Model 2	1.00	0.52 (0.37–0.73)	0.39 (0.26–0.58)	0.0001	0.83 (0.77–0.90)	1.00	0.64 (0.46–0.90)	0.48 (0.26–0.90)	0.015	0.68 (0.50–0.92)
Model 3	1.00	0.50 (0.36–0.70)	0.35 (0.24–0.52)	<0.0001	0.82 (0.76–0.88)	1.00	0.45 (0.31–0.65)	0.30 (0.17–0.54)	0.0001	0.52 (0.39–0.70)
Development set										
Deceased/survival, n	78/32	69/101	41/153			85/72	53/134	24/103		
Years of follow-up, median	5.75	8.71	8.95			8.17	8.95	8.93		
Cumulative death rate, per 1,000 personal year	130	56.5	25.8			83.5	35.3	22.8		
Crude model	1.00	0.31 (0.20–0.48)	0.15 (0.09–0.26)	<0.0001	0.69 (0.64–0.75)	1.00	0.37 (0.25–0.56)	0.20 (0.11–0.35)	<0.0001	0.78 (0.72–0.84)
Model 1	1.00	0.33 (0.21–0.50)	0.18 (0.11–0.29)	<0.0001	0.71 (0.65–0.77)	1.00	0.52 (0.33–0.81)	0.30 (0.13–0.65)	0.003	0.82 (0.75–0.90)
Model 2	1.00	0.39 (0.27–0.58)	0.20 (0.12–0.35)	<0.0001	0.73 (0.66–0.80)	1.00	0.51 (0.33–0.78)	0.28 (0.12–0.66)	0.001	0.82 (0.75–0.89)
Model 3	1.00	0.39 (0.26–0.59)	0.18 (0.10–0.32)	<0.0001	0.72 (0.65–0.79)	1.00	0.38 (0.26–0.56)	0.20 (0.11–0.38)	<0.0001	0.77 (0.73–0.83)
Validation set										
Deceased/survival, n	66/48	66/104	68/131			69/69	54/131	27/120		
Years of follow-up, median	6.72	8.73	8.76			8.33	8.86	8.98		
Cumulative death rate, per 1,000 personal year	96.4	53.2	44.4			78.3	37.7	22.0		
Crude model	1.00	0.55 (0.38–0.80)	0.45 (0.32–0.64)	0.0002	0.86 (0.80–0.92)	1.00	0.44 (0.28–0.71)	0.23 (0.12–0.42)	<0.0001	0.81 (0.75–0.86)
Model 1	1.00	0.61 (0.41–0.89)	0.52 (0.35–0.75)	0.002	0.89 (0.82–0.96)	1.00	0.79 (0.44–1.42)	0.48 (0.23–1.02)	0.065	0.89 (0.82–0.96)
Model 2	1.00	0.57 (0.37–0.86)	0.61 (0.38–0.97)	0.068	0.92 (0.85–1.01)	1.00	0.92 (0.46–1.82)	0.72 (0.30–1.73)	0.475	0.92 (0.83–1.02)
Model 3	1.00	0.56 (0.37–0.84)	0.56 (0.35–0.90)	0.033	0.91 (0.83–0.99)	1.00	0.56 (0.31–1.01)	0.39 (0.18–0.87)	0.023	0.85 (0.77–0.94)

Data were weighted for unequal probability of sampling design by SUDAAN. Hazard ratios were estimated by the Cox proportional hazard model.

Model 1: adjusted for age. Model 2: adjusted for age, region, education level, smoking status, physical activity, social engagement, and cognitive impairment.

Model 3: adjusted for region, education level, smoking status, physical activity, social engagement, and cognitive impairment.

HANI, Healthy Ageing Nutrition Index; NAHSIT, Nutrition and Health Survey in Taiwan.

Women with HANIs of 14–20 and >20 exhibited a lower risk of all-cause mortality in the development set (HR: 0.37, 95% CI: 0.25–0.56; HR: 0.20, 95% CI: 0.11–0.35) and the validation set (HR: 0.44, 95% CI: 0.28–0.71; HR: 0.23, 95% CI: 0.12–0.42), respectively (both *P* for trend <0.0001). However, the magnitude for this trend was reduced after adjustment for age in the validation set. With full adjustment (model 3), the risk of mortality was reduced by 18% and 8% when HANI increased by 2.

**Figure 3:**
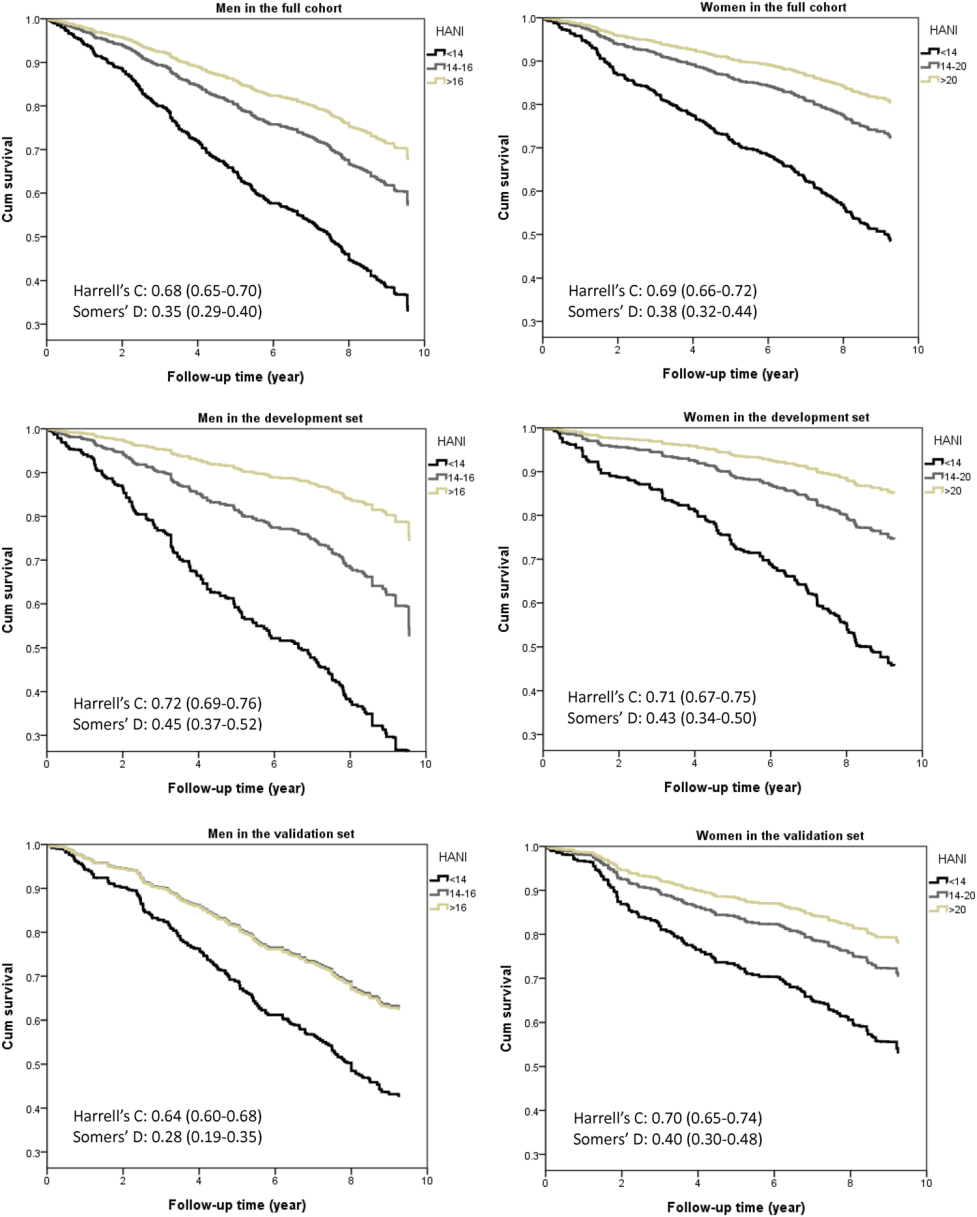
Cumulative survival curves for all-cause mortality by HANI, adjusted for region, education level, smoking status, physical activity, social engagement, and cognitive impairment. HANI, Healthy Ageing Nutrition Index.

For the entire cohort, the HRs for men with HANIs of 14–16 and >16 were 0.50 (0.36–0.70) and 0.35 (0.24–0.52), respectively, compared with those with HANI < 14. Women with the highest HANI exhibited a 70% lower mortality risk (HR: 0.30, 95% CI: 0.17–0.54). The entire study cohort behaved in the same manner as the development set, as shown in the survival curves in Fig. 3. There was equally good discrimination between the development and validation sets. Men in the validation set had a similar and overlapping cumulative survival rate between HANI 14–16 and that greater than 16. This is confirmed by comparable Harrell’s C and Somers’ D scores, more so for women than men.

## Discussion

It is possible to identify factors that are non-invasively obtained and are potentially modifiable nutrition-related predictors of survival. The gender-specific and composite indices for survival prediction were the dietary pattern, food preparation, social circumstances of eating, and body composition. Confidence in HANI was gained by the use of predictive power statistics applied to both development and validation data sets. Moreover, the predictability of HANI is superior to that of any of its individual components.

The nutrition-related factors that are mainly associated with reduced survival are less PA^10^, loss of appetite^21^, chronic energy deficiency^30^, weight loss^31^, and sarcopenia^32,33^, and all are interconnected. In a Taiwanese cohort, regular moderate PA for 30–40 min daily was associated with reduced mortality^10^. However, PA did not modify the association between HANI and survival, suggesting that there may be another means of formulating a predictor of survivorship. In this study, we may have captured potentially predictive variables like PA and frailty by the inclusion of others like eating alone, cooking or BMI.

The Mini Nutritional Assessment (MNA) is used to detect institutional undernutrition; in its short-form (6 items) it is not associated with mortality, although each item alone is associated with mortality in older free-living women^34^. In HANI for men, loss of appetite, as detected by the MNA, is an individual predictor of mortality. The Geriatric Nutritional Risk Index comprises weight, weight loss, and albumin which limits its use in community settings because of the dependence on memory and the need to obtain biomarkers^35^. In HANI, the factors chosen (including eating alone, food choice, and cooking) may represent the earlier development of risk and the higher likelihood of effective intervention. Seniors in the Community: Risk Evaluation for Eating and Nutrition, version II, has been validated for Canadian older people. This index includes 17 items that are similar to those in HANI which can be used in community settings for nutritional screening^36^. However, the predictive ability of this index for disability, disease, and mortality is unclear.

In HANI, DDS and BMI are common to both genders by analysis and deduction. Encouragement to improve dietary quality exerts favourable effects on food patterns and the intake of nutritious food components. The shared gender relevance of a diverse diet in survival is evident^19,37^. DDS was measured by a 24-hr dietary recall in this study. It can be rapidly assessed by asking participants if they consumed half a serving size of each food group on the previous day (such information can be inserted into the online HANI app https://ychuang.shinyapps.io/HANI/, Supplementary Fig. S1). This can be applied in both community and clinical settings for nutritional evaluation and education.

The finding presumably reflects the importance of adequate energy throughput to the achievement of an adequate intake of favourable food components (possibly with more fat mass), along with preservation of muscle (less sarcopenia). As in other studies, we identified a higher BMI to be a survival advantage. The waist circumference was not an independent survival predictor. Sarcopenia increases with age and is often obscured by increasing body fat^33^. Along with reduced muscle strength, it contributes to frailty and mortality. Similar to sarcopenia, SMMI is a predictor of survival in this population^32^ and positively associated with HANI. SMMI has been taken into account for this population, used in the cross-validation of HANI, but are not included in the indices as items which are less routinely available.

Good appetite was a survival advantage in men, but not in women. The gender difference may be based on the relative inability of men to maintain healthy dietary and PA practices, which encourage a more appropriate appetite with age. The corollary in women, to their advantage, would be that more frequent cooking contributes substantially to the association of HANI with survival^18,38^. We hypothesize that the findings related to the predictive ability of HANI for survival (i.e., appetite and eating alone in men and cooking in women) are linked. Anorexia associated with ageing and loss of desire to eat are contributors to poor nutritional status^21^. Pathophysiologic anorexia associated with ageing develops when there is failure to regulate food intake adequately^39,40^. In the NHANES III of America, food intake decreases linearly by age and is probably associated with reduced PA and energy metabolism^40^. However, increased energy intake was associated with increasing HANIs in men (*P* = 0.006, data not shown), but not in women. This finding suggests that energy intake and higher HANI are more dependent on appetite in men. A possible mechanism is that testosterone levels decline with age and are inversely associated with leptin levels, thus leading to diminished food intake and an increased metabolic rate^41,42^.

Cooking and social engagement may affect both the quality and quantity of food intake^24^. We identified cooking to be a predictor of HANI only in women. Women usually prepare meals in Taiwan. The cooking involved requires at least planning, food choice, meal preparation itself, and various PAs^18,38^. Women deal with life alone more favourably than men^43^. The highest HANI participants and being more likely to live alone were women. By contrast, those registering the lowest HANI and living alone were men. Men tended to eat with others or consume fast food when they lived alone. Hence, eating with others seems crucial for men^44^. It may reduce the risk of malnutrition through social support, more food variety with improved dietary quality as well as QOL^45^. Altogether, cooking and eating with others may have combined benefits for nutritional status; these benefits are indicative of socio-psychological factors and therefore contribute to survival. The tangible support derived from eating with others has been associated with increased fruit and vegetable intakes in older men. Among women, emotional and informational support increased these intakes. By contrast, women have healthier diets when they cook for themselves^29^. This supports cooking as associated with more benefits for women and that eating with others is crucial for men.

We did not find the expected associations between HANI and cardiometabolic risk factors. Men with the highest HANI had higher triglycerides and diastolic blood pressure, and women with the highest HANI had higher total and LDL cholesterol. A lower cholesterol is associated not only with malnutrition, but also increased mortality in elders^46,47^. The advantage of HANI is that its predictive ability does not depend on cardiometabolic risk factors or detailed body compositional analysis^32^.

Our study has some strengths and limitations. First, we demonstrate that composite nutritionally modifiable factors, characterized by engagement and the ultimate consumption of a diversified diet, are conducive to higher survival. HANI, in this particular study context, may be a surrogate for associated health-promoting factors. Although we adjusted for several health domains, residual confounders likely remain, and HANI is unlikely to have a cause-and-effect relevance on its own. It must be modified if the community under investigation is socioculturally different. Second, it is both a limitation and a strength that HANI is gender-specific, a finding that demonstrates differences between older men and women. Third, people with a history of chronic diseases at baseline were not excluded. However, dietary habits that may change owing to disease have not been considered. It must be emphasized that the representative study population was free-living in the community, which means participants were functionally healthy. For policy relevance, community-based elderly have been the focus of our investigation. Nevertheless, the final indices did not change in the model adjusted for multi-morbidity (Charlson comorbidity index, Supplementary Table S2). Fourth, when we claim non-invasive assessment for the factors in HANI, we made anthropometric measurements to calculate BMI. Fifth, we examined the gender-specific indices with and without age for the development and validation sets and the entire study cohort. For women, after adjustment for age, the validation is less predictive of survival. This might be attributed to an over adjustment for age (twice: before and in the model). Another reason might be that the very old women cooked less, leading to a drop in their sample size and to statistical instability. Another consideration is that women outlive men and may represent an increasingly sociobiologically heterogeneous group with advancing years.

For senior citizens, HANI can offer a modifiable predictor of survival that is accessible, socioculturally adaptable, gender-specific, and may alter outcomes, although this would appear context-dependent. It comprises appetite, eating with others, dietary diversity score, and BMI for men, and cooking frequency, dietary diversity score, and BMI for women. The utility of HANI for the older population studied can be enhanced by the provision of an online assessment and monitoring tool. This tool can be used for several purposes such as nutritional education in the community or general population, and diagnosis of potential risk of nutritional disorder for further intervention in clinical settings. HANI is available for aged care policy makers and workers.

## Methods

**Figure 4:**
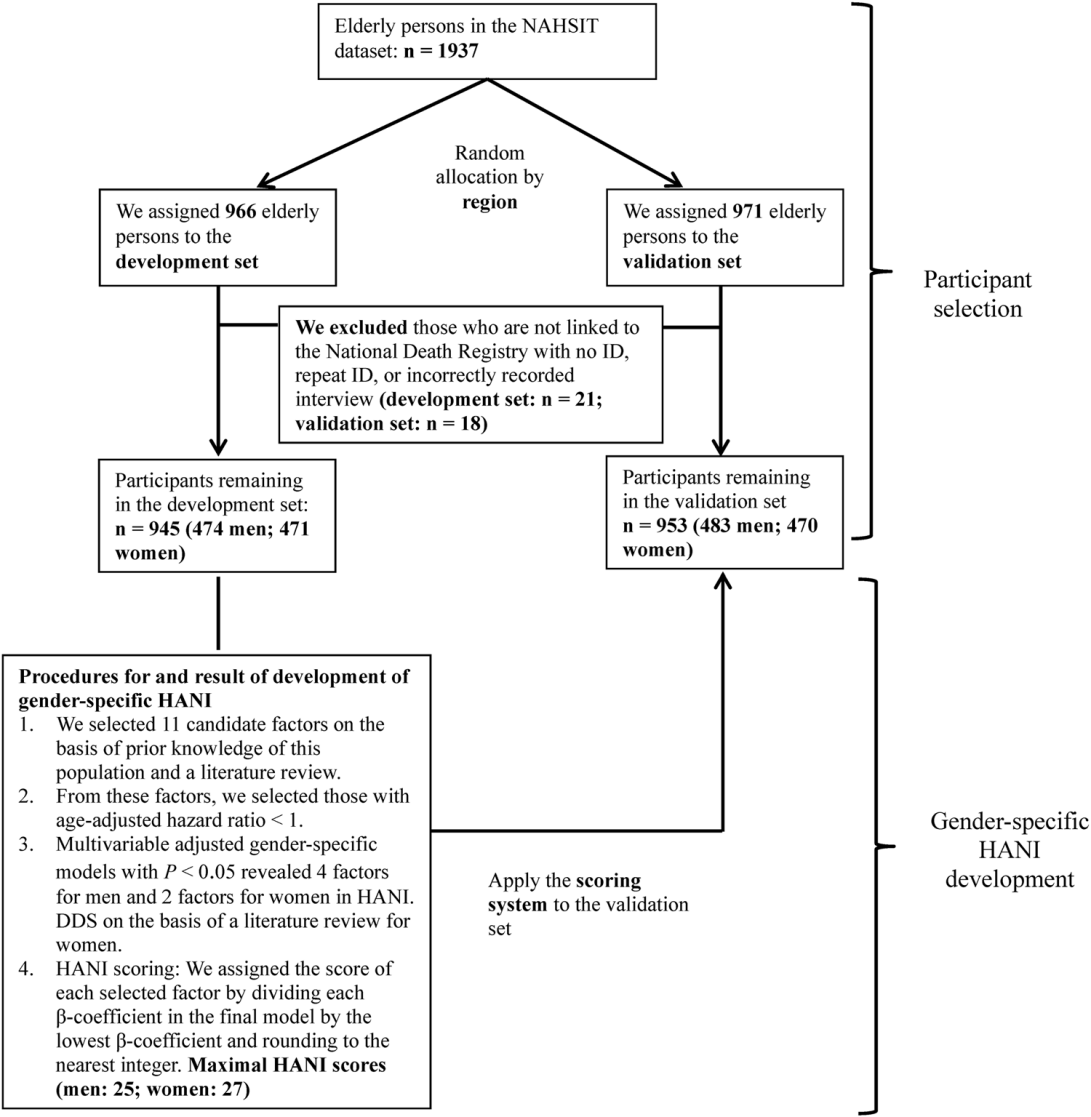
Flow chart of participant selection and procedure for the development of gender-specific HANI. NAHSIT, Nutrition and Health Survey in Taiwan; HANI, Healthy Ageing Nutrition Index; DDS, dietary diversity score.

Cross-sectional and prospective study designs were used to evaluate the utility and validity of HANI. We recruited participants from the 1999–2000 Nutrition and Health Survey in Taiwan (NAHSIT). A total of 1937 older people aged 65 y or older completed face-to-face interviews. We randomly divided the participants by region into development (n = 966) and validation (n = 971) sets (Fig. 4). The distributions of the two datasets are shown in Supplementary Table S3. At the baseline, participants provided information through a self-reported questionnaire and underwent a physical examination in the morning. We collected their fasting blood for metabolic profiling. Informed consent was obtained from participants at interview. Ethical approval was obtained from the Institutional Review Boards of the National Health Research Institutes and Academic Sinica, Taiwan.

### Nutritionally modifiable factors

We selected 11 nutritionally modifiable factors predicting mortality through prior knowledge of this population and a literature review. They were appetite^21^, chewing ability^22^, DDS^19^, daily vegetable expenditure^48^, frequency of cooking^18^, frequency of eating with others^44^, frequency of shopping^16^, alcohol consumption^49^, PA^10^, BMI^50^, and waist circumference^51^. The measurement details for 11 candidate factors are summarized in Table 1.

Dietary information was obtained through a one day 24-h dietary recall and a simplified food frequency questionnaire. Dietary quality was assessed using DDS based on a half serving of 6 food groups a day. The DDS ranged from 0 to 6, with a higher score representing better dietary quality^19^. We calculated participants’ daily vegetable expenditure by using 24-h dietary recall^52^. Cut-off points for BMI and waist circumference were in accord with Taiwanese recommendations^53^. Participants with BMI (kg/m^2^) <18.5 were considered underweight, 18.5–23.9 as normal weight, 24–26.9 as overweight, and ≥27 as obese. We defined normal waist circumference as waist circumferences of <90 and <80 cm in men and women, respectively. We compiled the measure of PA as metabolic equivalents (METs) per day. We classified the participants into 3 groups on the basis of daily METs: <1.5 (moderate PA < 30 min), 1.5–3 (moderate PA 30–60 min or vigorous PA < 30 min), and >3 (moderate PA ≥ 60 min or vigorous PA ≥ 30 min)^54^.

### Other contextual factors

We measured health-related QOL by using the Chinese version of the Short Form (SF-36®), modified for Taiwanese, which contains 36 self-assessment questions to measure 8 dimensions of health following the norm-based scoring system (μ = 50, σ = 10)^55^. A higher score indicates better QOL.

Cognitive impairment was assessed by a validated Short Portable Mental Status Questionnaire (SPMSQ) in Chinese. A total of 10 questions regarding orientation in time and place, personal history, long-term and short-term memory and calculation was use to evaluate mental health. Cognitive impairment was defined as ≥3 errors in the answers to the questions. Social engagement was assessed by 3 questions about visiting relatives, engaging in religious activities and involvement in social activities. ‘Less social engagement’ was defined as never being involved in these activities.

Indicators of muscle mass included SMMI, TSF, MAC, and MAMC. We assessed sarcopenia by calculating the SMMI by the following equation^32^:

$${\rm{SMMI}}=[0.401\times ({{\rm{height}}}^{2}/{\rm{resistance}})+(3.825\times {\rm{gender}})-(0.071\times {\rm{age}})+5.102]/{{\rm{height}}}^{2}$$

where height is measured in meters, resistance in Ohms, and age in years; men = 1 and women = 0. Resistance for whole body SMM was assessed by a BIA device (Parama-Tech BF-101) with two electrical signals (right wrist and right ankle). The equation was developed by Janssen *et al*.^56^ and validated for Taiwanese elders by MRI-measured skeletal muscle mass^57^. The distribution of SMMI and correlation coefficients with other indicators of muscle mass are shown in the Supplementary Table S4.

$${\rm{We}}\,{\rm{calculated}}\,{\rm{MAMC}}\,{\rm{as}}\,{\rm{MAMC}}\,({\rm{cm}})={\rm{MAC}}({\rm{cm}})-(\pi \times {\rm{TSF}}({\rm{cm}})).$$

### Outcome ascertainment

The National Death Registry database was obtained from the Department of Health, Executive Yuan. NAHSIT data were linked to this database by IDs to determine survival. All deaths between the baseline of 1999–2000 and December 31, 2008, were counted.

### Healthy Ageing Nutrition Index (HANI)

We evaluated the association between the 11 candidate factors and all-cause mortality in the development set by using to step-by-step Cox proportional-hazard regression, in the following sequence (Fig. 4):

1. **Candidate factor selection** (refer to earlier text).
2. **Age-adjusted hazard ratio:** We determined the age-adjusted HR of each candidate factor. We selected the factors with age-adjusted HRs <1 for men and women.
3. **Identified factors in a composite survival index:** We entered these factors into a multivariable Cox proportional-hazard model manually to identify these factors according to their *P* values.
4. **HANI scoring:** We assigned the score of each selected factor by dividing each β-coefficient in the final model by the lowest β-coefficient and rounding to the nearest integer. We assigned HANI to each participant and summed the scores for all factors presented.
5. **Cut-points:** The cut-points for HANI was determined by the Youden index^58^.

### Statistical analysis

All analyses were stratified by gender, by using SAS software (v 9.1.3, SAS Institute Inc.), SUDAAN software (v 9.0, Research Triangle Institute), STATA MP 14 (Stata, College Station, TX) and R software (v3.4.0). Continuous and categorical variables are expressed as means ± standard errors (SEs) and percentages, respectively. We evaluated the corresponding differences by ANOVA and chi-square test. Missing values in this study were principally due to survey design where two data collection activities, household questionnaire interview (n = 1937) and physical check-up (n = 2432), were combined and not always congruent. In order to achieve study power and not to over-estimate effects, we imputed missing data for candidate factors as the poorest group for categorical variables or mean for BMI and waist circumference in the same age by year and gender group. The distributions of each candidate factor with or without imputation in the development set by gender were not significantly different (Supplementary Table S5). The point estimates continued in the same direction after exclusion of participants with any missing HANI variable, significance disappeared, probably due to limited power (Supplementary Table S6).

The follow-up time was from the date of interview to either the date of death or December 31, 2008. We assessed the association between HANI and all-cause mortality by Cox proportional-hazards regression model. Covariates adjusted were adjusted for age (in year), region, education level (illiterate, some up to primary school, and high school and above), current smoking status (yes, no), PA (<1.5, 2.5–2.9, ≥3 METs/d), cognitive impairment (yes, no), and less social engagement (yes, no). Because PA and ADL are highly correlated, we did not adjust for ADL in the models to avoid collinearity.

To evaluate the predictability of each component of HANI and the HANI score, a time-dependent receiver operating characteristic curve (ROC) analysis was used to discriminate between death and survivorship. This analysis uses sensitivity and specificity, both of which are time-dependent, to measure the predictability of a survival model as measured by the AUC^59^. We used C-statistics by logistic regression to evaluate the predictive accuracy and Harrell’s C as well as Somers’ D statistics for discriminatory performance (predictive power) of survival models^60^.

### Data availability

The data that support the findings of this study are available from Academia Sinica and the Taiwan Department of Health, but restrictions apply to the availability of these data, which were used under license for the current study, and so are not publicly available. Data are however available from the authors upon reasonable request and with the permission of Academia Sinica and Taiwan Department of Health.

## Electronic supplementary material

Supplementary Tables and Figure
