# Supplementary material for: A non-invasive modifiable Healthy Ageing Nutrition Index (HANI) predicts longevity in free-living older Taiwanese
Source: Sci Rep. 2018 May 8;8:7113. doi: 10.1038/s41598-018-24625-3 (PMC5940774; doi:10.1038/s41598-018-24625-3)
Supplement: Supplementary file 1 — Supplementary Tables and Figure [file 41598_2018_24625_MOESM1_ESM.pdf]

A non-invasive modifiable Healthy Ageing Nutrition Index (HANI) predicts longevity in free-living older Taiwanese

Yi-Chen Huang PhD[1,2], Mark L. Wahlqvist MD[3,4,5], Yuan-Ting C. Lo PhD[4], Chin Lin PhD[4,6], Hsing-Yi Chang DrPH[3], Meei-Shyuan Lee DrPH[2,4,5*]

[1] Department of Nutrition, China Medical University, 91 Hsueh-shih Road, Taichung 40402, Taiwan, ROC

[2] Graduate Institute of Life Sciences, National Defense Medical Center, 161 Minchuan East Road, Sec. 6, Taipei, Taiwan 11490, ROC

[3] Institute of Population Health Sciences, National Health Research Institutes, 35 Keyan Road, Zhunan, Miaoli County 35053, Taiwan, ROC

[4] School of Public Health, National Defense Medical Center, 161 Minchuan East Road, Sec. 6, Taipei, Taiwan 11490, ROC

[5] Monash Asia Institute, Monash University, 900 Dandenong Road, Caulfield East, Melbourne, Victoria 3145, Australia

[6] Department of Research and Development, National Defense Medical Center, 161 Minchuan East Road, Sec. 6, Taipei, Taiwan 11490, ROC

Supplementary Figure S1. Webpage of online app (https://ychuang.shinyapps.io/HANI/).

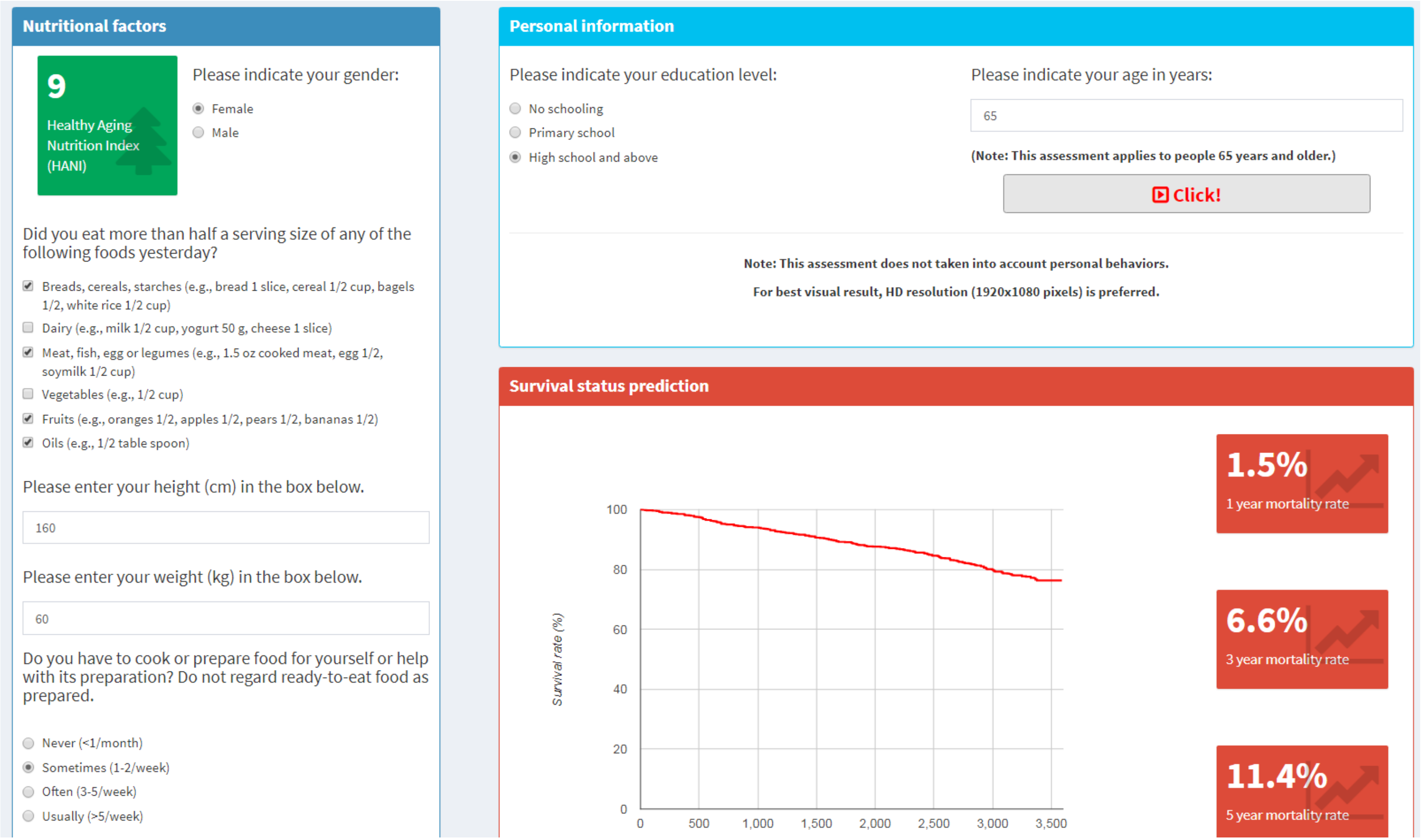

Supplementary Table S1. Food and nutrient intakes in NAHSIT Elderly by the HANI in the validation set.

	Men							Women						
	< 14		14–16		> 16		*P* value [a]	< 14		14–20		> 20		*P* value [a]
	Mean	SE	Mean	SE	Mean	SE		Mean	SE	Mean	SE	Mean	SE	
Food frequency intake (times/day)														
Dairy	0.46[a]	0.06	0.40[b]	0.06	0.82[ab]	0.07	<0.0001	0.72	0.12	0.83	0.11	0.85	0.07	0.624
Meat	1.26	0.14	1.27	0.13	1.38	0.14	0.768	0.98	0.12	0.98	0.13	0.94	0.08	0.920
Sea food	0.70[ab]	0.09	1.19[a]	0.16	1.00[b]	0.08	0.009	0.80	0.09	0.86	0.10	0.96	0.07	0.196
Egg	0.42	0.06	0.42	0.06	0.50	0.05	0.412	0.38	0.05	0.31	0.03	0.38	0.04	0.263
Soy	0.32	0.08	0.48	0.09	0.49	0.07	0.264	0.67	0.14	0.45	0.08	0.41	0.05	0.218
Vegetable	1.84[ab]	0.16	2.66[a]	0.19	2.54[ab]	0.12	0.002	2.45	0.19	2.28	0.15	2.44	0.16	0.525
Fruit	0.98[a]	0.12	0.92[b]	0.07	1.34[ab]	0.06	0.0004	0.87[a]	0.08	0.97[b]	0.07	1.43[ab]	0.09	<0.0001
Total energy intake, kcal	1534[a]	113	1704[b]	58.7	2067[ab]	122	0.001	1362	76.4	1384	117	1595	129	0.181
Protein, g/kg BW	1.11	0.14	1.38	0.09	1.32	0.08	0.249	1.26	0.08	1.05	0.07	1.19	0.11	0.055
Nutrient density, per 1,000 kcal														
Protein, g	43.6	3.04	43.8	1.82	43.1	1.07	0.932	41.9	1.51	42.0	1.60	44.6	1.24	0.195
Fat, g	31.1	1.87	29.0	1.71	30.0	1.35	0.629	27.5	1.35	27.5	2.00	27.3	1.35	0.993
Cholesterol, mg	173	20.8	135	10.3	142	11.0	0.260	130	14.4	111	7.25	125	7.63	0.311
Carbohydrate, g	133	4.88	140	4.86	140	3.52	0.452	147	3.79	147	4.73	146	2.52	0.979

Dietary fiber, g	10.8	0.74	11.5	0.81	12.7	0.49	0.061	12.0[a]	0.72	12.9	0.92	15.3[a]	0.80	0.008
Vit B-1, mg	0.71	0.12	0.62	0.04	0.83	0.07	0.031	0.72	0.04	0.66	0.04	0.76	0.06	0.237
Vit B-2, mg	0.93	0.14	0.71	0.06	0.94	0.06	0.050	0.77[a]	0.07	0.87[b]	0.06	1.16[ab]	0.07	0.001
Niacin, mg	10.4	1.05	9.40	0.71	8.97	0.29	0.346	8.00	0.35	8.17	0.45	8.82	0.33	0.199
Vit B-6, mg	0.90	0.08	0.78	0.06	0.69	0.03	0.100	0.70	0.04	0.70	0.05	0.79	0.05	0.293
Vit C, mg	76.1[a]	9.04	81.4[b]	8.90	100[ab]	6.61	0.015	79.5[a]	6.36	101[b]	9.91	144[ab]	17.1	0.002
Vit E, mg α-TE	5.39	0.50	4.83	0.45	4.56	0.27	0.404	4.86	0.31	6.15	0.91	4.93	0.32	0.395
Calcium, mg	318[a]	32.5	303[b]	22.1	441[ab]	27.2	0.002	403[a]	38.9	422[b]	32.3	572[ab]	35.8	0.002
Magnesium, mg	147	6.47	139	5.88	155	4.71	0.109	150[a]	7.65	148[b]	6.94	175[ab]	6.09	0.001
Iron, mg	7.79	0.50	8.04	0.60	7.20	0.28	0.369	7.53	0.54	7.88	0.52	8.30	0.68	0.626
Potassium, mg	1418	90.2	1459	108	1626	51.1	0.066	1425[a]	79.7	1514[b]	71.7	1746[ab]	57.2	0.002
Sodium, mg	3115	374	3097	376	2856	217	0.813	2753	282	3258	254	3067	313	0.424

All data are presented as mean and standard error (SE) and weighted for unequal probability of sampling design by SUDAAN. [a] We used ANOVA to test difference between the HANI groups by gender. The same superscript letter implies that there is *P* value less than 0.05 between the two groups by Bonferroni test. NAHSIT, Nutrition and Health Survey in Taiwan; HANI, Healthy Ageing Nutrition Index; SE, standard error; BW, body weight; Vit, vitamin; α-TE, α-tocopherol equivalent.

Supplementary Table S2. Hazard ratios (95% confidence interval) for the association between HANI and risk of all–cause mortality in NAHSIT Elderly by gender.

	HANI, hazard ratios (95% confidence interval)									
	Men					Women				
	< 14	14–16	> 16	*P* for trend	2 point increase	< 14	14–20	> 20	*P* for trend	2 point increase
Development set										
Final model	1.00	0.39 (0.26–0.59)	0.18 (0.10–0.32)	<0.0001	0.72 (0.65–0.79)	1.00	0.38 (0.26–0.56)	0.20 (0.11–0.38)	<0.0001	0.77 (0.73–0.83)
Further adjusted for CCI	1.00	0.39 (0.26-0.59)	0.18 (0.10-0.31)	<0.0001	0.72 (0.65-0.79)	1.00	0.37 (0.26-0.52)	0.19 (0.11-0.36)	<0.0001	0.77 (0.72-0.81)
Validation set										
Final model	1.00	0.56 (0.37–0.84)	0.56 (0.35–0.90)	0.033	0.91 (0.83–0.99)	1.00	0.56 (0.31–1.01)	0.39 (0.18–0.87)	0.023	0.85 (0.77–0.94)
Further adjusted for CCI	1.00	0.56 (0.38-0.84)	0.56 (0.36-0.87)	0.026	0.76 (0.60-0.96)	1.00	0.58 (0.31-1.05)	0.40 (0.18-0.90)	0.001	0.62 (0.41-0.95)

Data were weighted for unequal probability of sampling design by SUDAAN. Hazard ratios were estimated by the Cox proportional hazard model.
Final model: adjusted for region, education level, smoking status, physical activity, social engagement, and cognitive impairment.
HANI, Healthy Ageing Nutrition Index; NAHSIT, Nutrition and Health Survey in Taiwan; CCI, Charlson comorbidity index.

Supplementary Table S3. Distributions of the two datasets by gender

Variables	Men				Women			
	Total	< 14	14–16	> 16	Total	< 14	14–20	> 20
Development set								
n	474	110	170	194	471	157	187	127
Weight n	634739	134909	219948	279882	575446	219926	209615	145906
Total, %		21.2	34.7	44.1		38.2	36.4	25.4
Cumulative death rate, per 1,000 person-year		130	56.5	25.8		83.5	35.3	22.8
Validation set								
N	483	114	170	199	470	138	185	147
Weight n	632390	150700	219574	262116	588901	199495	200192	189215
Total, %		23.8	34.7	41.5		33.9	34.0	32.1
Cumulative death rate, per 1,000 person-year		96.4	53.2	44.4		78.3	37.8	22.0

Supplementary Table S4. Distribution of SMMI in validation set by gender and its correlation with other anthropometric measures.

	SMMI				Pearson correlation coefficient		
	Mean±SE	Median	Min	Max	TSF	MAC	MAMC
Men							
Crude	12.3±0.14	12.4	5.39	17.0	0.281	0.552	0.465
z score	-0.01±0.05	-3.73	2.51	2.51			
Women							
Crude	9.2±0.16	9.28	4.57	13.2	0.426	0.520	0.351
z score	-0.07±0.11	-0.042	-3.42	2.79			

SMMI, skeletal muscle mass index; TSF, triceps skinfold thickness; MAC, mid-arm circumference; MAMC, mid-arm muscle circumference.

Supplementary Table S5. Distributions of each candidate factor with or without imputation in the development set by gender

	Men				Women			
	Missing n	n with missing data (%)	n after imputation (%)	*P* value	Missing n	n with missing data (%)	n after imputation (%)	*P* value
Appetite status	12			0.199	12			0.323
Poor		26 (5.51)	38 (7.54)			47 (10.3)	59 (12.4)	
Fair		259 (52.6)	259 (51.5)			296 (60.4)	296 (59.0)	
Good		183 (41.9)	183 (41.0)			131 (29.3)	131 (28.6)	
Satisfactory chewing	14	169 (35.2)	183 (37.0)	0.264	12	197 (42.3)	209 (43.9)	0.335
Dietary diversity score	7			0.821	8			0.823
≤ 3		82 (14.3)	89 (15.5)			93 (19.0)	101 (20.3)	
4		152 (30.8)	152 (30.4)			164 (30.3)	164 (29.8)	
5		166 (37.1)	166 (36.6)			156 (35.2)	156 (34.7)	
6		73 (17.7)	73 (17.5)			65 (15.4)	65 (15.2)	
Vegetable expenditure, NTD/day	52			0.274	35			0.203
< 10.07		110 (22.6)	129 (29.4)			123 (26.0)	110 (24.0)	
10.07–< 18.76		115 (28.5)	119 (28.6)			138 (30.8)	116 (25.5)	
18.76–< 26.05		76 (20.3)	122 (19.0)			86 (19.0)	130 (21.0)	
≥ 28.05		127 (28.6)	110 (23.1)			104 (24.2)	130 (29.5)	
Cooking frequency				1.000	3			0.999
Never (< 1/month)		259 (56.7)	259 (56.7)			103 (28.0)	106 (28.2)	

Sometimes		103 (20.6)	103 (20.6)			73 (13.1)	73 (13.1)	
Often		49 (8.40)	49 (8.40)			60 (10.3)	60 (10.3)	
Usually		69 (14.)	69 (14.)			247 (48.6)	247 (48.5)	
Eat with others	2	404 (86.7)	404 (86.4)	0.824	0	396 (81.9)	396 (81.9)	1.000
Alcohol drinking	1	163 (29.8)	164 (29.9)	0.945	2	39 (5.49)	41 (5.78)	0.687
Shopping frequency	11			0.959	10			0.994
< 1/week		209 (42.2)	218 (43.0)			234 (51.8)	241 (51.8)	
1/week		65 (13.0)	65 (12.7)			90 (16.4)	90 (16.2)	
2–4/week		102 (22.1)	104 (22.1)			100 (20.5)	102 (20.7)	
Everyday		93 (22.7)	93 (22.2)			52 (11.3)	53 (11.4)	
Physical activity, METs/day	6			0.972	9			0.964
< 1.5		261 (48.9)	263 (48.6)			311 (60.9)	319 (61.3)	
1.5–2.9		59 (11.5)	59 (11.3)			54 (11.9)	54 (11.7)	
≥ 3		154 (36.7)	158 (40.1)			112 (27.2)	113 (27.0)	
BMI, kg/m^2	129	23.4±0.18	23.4±0.14	0.542	113	23.8±0.30	23.8±0.21	0.677
waist circumference	131	85.9±0.44	85.7±0.32	0.609	117	81.5±0.66	81.4±0.48	0.659

Supplementary Table S6. Hazard ratios (95% confidence interval) of data with or without imputation for the association between HANI and risk of all–cause mortality in NAHSIT Elderly by gender.

	HANI, hazard ratios (95% confidence interval)							
	Men				Women			
	< 14	14–16	> 16	*P* for trend	< 14	14–20	> 20	*P* for trend
Development set								
Final model	1.00	0.39 (0.26–0.59)	0.18 (0.10–0.32)	<0.0001	1.00	0.38 (0.26–0.56)	0.20 (0.11–0.38)	<0.0001
No imputation	1.00	0.99 (0.64–1.53)	0.72 (0.41–1.26)	0.290	1.00	0.65 (0.38–1.10)	0.28 (0.11–0.70)	0.006
Validation set								
Final model	1.00	0.56 (0.37–0.84)	0.56 (0.35–0.90)	0.033	1.00	0.56 (0.31–1.01)	0.39 (0.18–0.87)	0.023
No imputation	1.00	0.71 (0.43–1.15)	0.67 (0.46–0.97)	0.014	1.00	0.55 (0.26–1.16)	0.42 (0.17–1.06)	0.068

Data were weighted for unequal probability of sampling design by SUDAAN. Hazard ratios were estimated by the Cox proportional hazard model.

Final model: adjusted for region, education level, smoking status, physical activity, social engagement, and cognitive impairment.

HANI, Healthy Ageing Nutrition Index; NAHSIT, Nutrition and Health Survey in Taiwan.
